# Metastatic breast cancer cells in lymph nodes increase nodal collagen density

**DOI:** 10.1038/srep10002

**Published:** 2015-05-07

**Authors:** Asif Rizwan, Camille Bulte, Anusha Kalaichelvan, Menglin Cheng, Balaji Krishnamachary, Zaver M. Bhujwalla, Lu Jiang, Kristine Glunde

**Affiliations:** 1 The Johns Hopkins University In Vivo Cellular and Molecular Imaging Center, Division of Cancer Imaging Research, The Russell H. Morgan Department of Radiology and Radiological Science, The Johns Hopkins University School of Medicine, Baltimore, Maryland; 2 Department of Neuroscience, College of Literature, Science, and the Arts Honors Program, University of Michigan – Ann Arbor, Michigan; 3 Department of Health Sciences, Western University, London, Ontario, Canada; 4 The Johns Hopkins University School of Medicine, The Sidney Kimmel Comprehensive Cancer Center, Baltimore, Maryland

## Abstract

The most life-threatening aspect of breast cancer is the occurrence of metastatic disease. The tumor draining lymph nodes typically are the first sites of metastasis in breast cancer. Collagen I fibers and the extracellular matrix have been implicated in breast cancer to form avenues for metastasis. In this study, we have investigated extracellular matrix molecules such as collagen I fibers in the lymph nodes of mice bearing orthotopic human breast cancer xenografts. The lymph nodes in mice with metastatic MDA-MB-231 and SUM159 tumor xenografts and tumor xenografts grown from circulating tumor cell lines displayed an increased collagen I density compared to mice with no tumor and mice with non-metastatic T-47D and MCF-7 tumor xenografts. These results suggest that cancer cells that have metastasized to the lymph nodes can modify the extracellular matrix components of these lymph nodes. Clinically, collagen density in the lymph nodes may be a good marker for identifying lymph nodes that have been invaded by breast cancer cells.

The American Cancer Society's estimates that there will be about 232,570 new cases of invasive breast cancer and about 40,000 deaths from breast cancer in the USA in 2014^1^. The occurrence of metastasis is the most life-threatening aspect of breast cancer^1^. Cancer cells that break away from the primary tumor can invade the circulation through either the blood vessels or lymphatic vessels (Supplementary Figure 1). Throughout the body, excess extravascular fluid passes through lymph nodes and is returned to the venous system^2^. Thus, lymph nodes get exposed to any tumor cells present in the lymphatic vessels^3^, and lymph nodes in the proximity of the primary tumor that are draining it have an increased chance of developing a secondary tumor^4^,^5^. In addition, lymph nodes also have their own vascularization, so that it is possible for circulating tumor cells to invade lymph nodes by escaping from blood vessels as well^5^,^6^.

The lymphatic vessels of the breast drain primarily into the axillary lymph nodes^7^. The lymphatic drainage of the breast is of great importance in the spread of metastatic breast cancers^8^. Patients with four or more positive, cancer cell containing lymph nodes have a significantly worse outcome regardless of the duration of the disease-free interval^9^. Recent research suggests an interaction between cancer cells and the lymphatic endothelium by which cancer cells may travel from the primary tumor site towards the lymph nodes^10^. Using lymphatic vessel specific markers, intratumoral lymphatic vessels have been observed in several types of tumors^11^,^12^,^13^. Quantitative fluorescence microscopy in breast cancer xenograft models showed that increased lymph node metastasis is correlated with enhanced invasiveness of cancer cells and a reduced tumor extracellular matrix (ECM) integrity^14^.

The first lymph nodes into which lymphatic fluid is drained from a primary breast tumor are the sentinel lymph nodes, and as a consequence, they are typically the first lymph nodes to contain cancer cells that have escaped from the primary tumor. During sentinel lymph node biopsy, a tracer molecule is injected near the tumor that helps the surgeon to identify and remove the sentinel lymph nodes for further analysis. Metastatic tumors can alter the organization of venous blood vessels and can increase the proliferation of endothelial cells within the sentinel lymph nodes^15^. This results in a functional shift of the venous blood vessels in lymph nodes from immune response mediator to blood flow carrier, and thereby increases nutrient and oxygen supply to the metastatic breast cancer cells in the lymph node^16^.

By following a sequential progression, breast cancer cells arrive at the sentinel lymph nodes and from there they invade neighboring lymph nodes. If sentinel nodes are not affected by metastases, it is typically unlikely that any of the other lymph nodes are affected by the cancer^17^. Human tissue microarrays revealed a higher presence of ECM molecules such as fibronectin, laminin, galectin-3, and galectin-8 in sentinel lymph nodes of breast cancer patients with malignant disease compared to those without cancer^18^. A separate clinical study of fifty breast cancer patients and 34 healthy controls showed higher levels of gelatinase-A (MMP-2) and gelatinase-B (MMP-9) in sentinel lymph nodes containing macroscopic metastatic nodules with respect to lymph nodes that were free of cancer cells^19^. An increased number of macrophages was observed in the draining lymph nodes of breast cancer patients, in which these macrophages were shown to phagocytose keratinic debris^20^.

In this study, we have identified distinct changes in the ECM components of lymph nodes, in particular in their collagen fiber matrix, in response to the presence of metastatic breast cancer cells in the lymph nodes of mouse models of breast cancer. Sentinel lymph nodes in mice growing metastatic human breast tumor xenografts, or growing xenografts generated from circulating tumor cells (CTCs), exhibited increased collagen density and increased periodic acid – Schiff (PAS) positive basement membrane, polysaccharides, and mucosubstances.

## Results

### Cytokeratin positive cancer cells in the lymph nodes of metastatic breast tumor models

**Figure 1:**
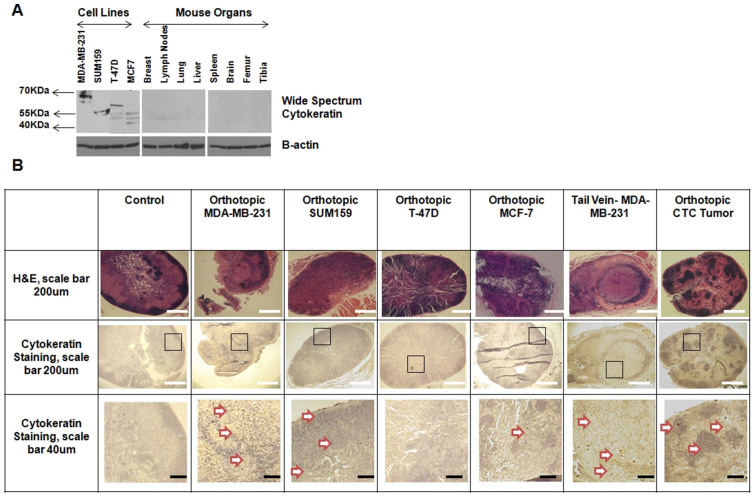
Metastatic colonies identified in the lymph nodes of mice growing metastatic tumor xenografts or tail vein injected with metastatic tumor cells. (A) Immunoblot showing that the breast cancer cell lines used in this study highly express several cytokeratins, whereas metastatic target organs do not. (B) Positive immunoperoxidase stain for cytokeratins was evident in lymph nodes from mice with orthotopic MDA-MB-231 and SUM159 tumors, tail vein injected MDA-MB-231 cells, and orthotopic MDA-MB-231-derived CTC tumors. No stain was observed in lymph nodes from mice with orthotopic T-47D and MCF-7 tumors. White scale bars are 200 μm and black scale bars are 40 μm.

Immunoblots for a wide spectrum of different cytokeratins with varying molecular weights of 58, 56, 52, 60, 51, 48, and 68 kDa was performed for all breast cancer cell lines and control organs as shown in Figure 1A. Cytokeratins were present in all breast cancer cell lines, but not in any organs (Figure 1A). In order to verify the presence of metastatic breast cancer cells in lymph nodes, we performed an immunoperoxidase stain for cytokeratin on the lymph nodes from all animal models. Cytokeratin positive, metastasized cancer cell containing areas in the lymph nodes were identified by dark brown color in Figure 1B and are pointed out by arrows. The lymph nodes from the metastatic MDA-MB-231 and SUM159 xenografted mice, as well as mice that were injected with MDA-MB-231 cells through the tail vein, or xenografted with MDA-MB-231-derived CTCs, contained cytokeratin, visualizing cancer cells that had metastasized to these lymph nodes. The number of cytokeratin positive cancer cells in the lymph nodes of all metastatic groups is in the tens, and not in the hundreds. Therefore, they can be clearly detected at higher magnification. In contrast, lymph nodes from control mice and non-metastatic T47D showed no presence of cytokeratin (Figure 1B). The number of cytokeratin positive cancer cells in the lymph nodes of mice growing non-metastatic MCF-7 tumors is significantly lower than that of the metastatic groups and hence can only be observed at very high (400X) magnification.

The hematoxylin and eosin (H&E) stains of lymph nodes from mice growing metastatic SUM159 tumor xenografts (Figure 1B) and mice where MDA-MB-231 cells were injected through the tail vein (Figure 1B), show a lack of B cell rich follicles, compared to lymph nodes from other groups.

### Increased collagen density in the lymph nodes of metastatic breast tumor models

**Figure 2:**
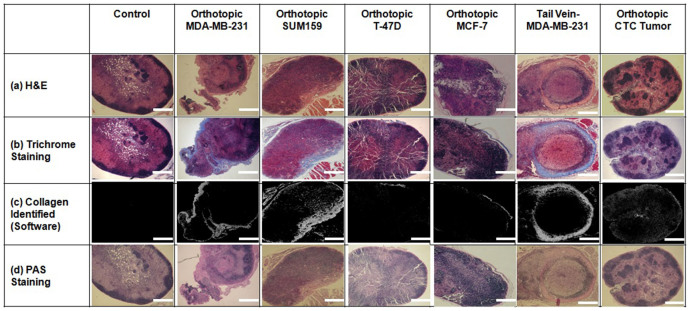
Collagen fiber density increases in lymph nodes of mice with metastatic primary tumors. Representative images are shown from H&E staining (top row), Masson's Trichrome staining (second row), collagen fibers identified by Masson's Trichrome staining (third row), and PAS staining (last row) of lymph nodes from all experimental groups. Scale bars are 200 μm.

**Figure 3:**
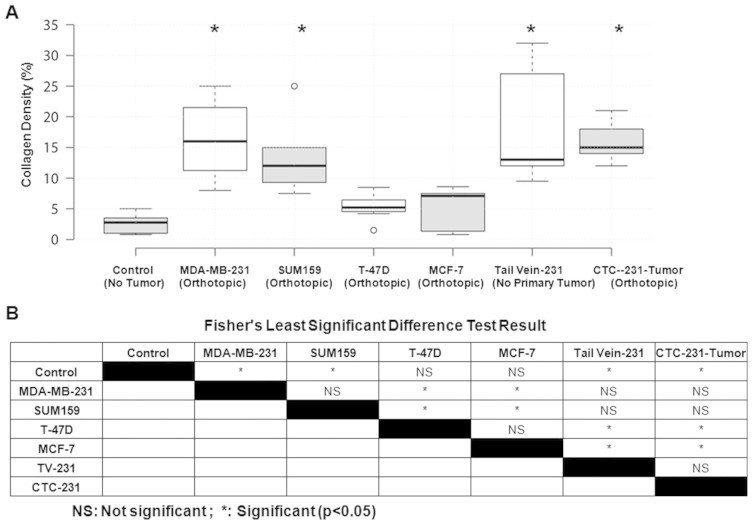
Quantification of collagen fiber density in lymph nodes. (A) The boxplots represent the collagen fiber density quantified in the lymph nodes of mice with no tumor (control, n = 5), and orthotopic MDA-MB-231 (n = 4), SUM159 (n = 3), T-47D (n = 4), MCF-7 (n = 3) tumors, tail vein injected MDA-MB-231 cells (n = 3), and orthotopically grown MDA-MB-231-derived CTC tumors (n = 3). Center lines show the medians, box limits indicate the 25th and 75th percentiles as determined by the R software, whiskers extend 1.5 times the interquartile range from the 25th and 75th percentiles, and outliers are represented by dots. (B) Following one-way analysis of variance (ANOVA) of collagen fiber densities, Fisher's Least Significant Difference (LSD) was calculated to compare the mean of one group with the mean of another group. p < 0.05 was considered statistically significant and is indicated by *.

The collagen fiber density in lymph nodes from mice bearing metastatic MDA-MB-231 and SUM159 tumors was significantly increased compared to that of control mice bearing no tumor, as well as compared to that of mice growing non-metastatic T-47D and MCF-7 tumors (Figure 2). Collagen fibers are shown in blue in the Trichrome stained images. Similarly, we also observed PAS positive, magenta-colored positive polysaccharides, mucosubstances, and basement membrane in lymph nodes from the metastatic xenograft models, especially in the regions where the density of Trichrome stained collagen was high. The lymph nodes of mice that had been injected with MDA-MB-231 tumor cells into their tail veins contained higher collagen densities than those of control mice and non-metastatic breast tumor xenograft models. In some cases of tail vein injected mice, the collagen fibers in the lymph nodes were arranged in a circular pattern that surrounded regions where cytokeratin-positive MDA-MB-231 cells had metastasized. This circular arrangement of collagen also co-localized with an increased amount of PAS positive magenta, as shown in the PAS stain in Figure 2. We also investigated tumor xenografts that were orthotopically grown from MDA-MB-231 derived CTCs. Collagen fiber density was increased in the lymph nodes of mice growing CTC-derived tumor xenografts as compared to lymph nodes from control mice and mice with non-metastatic tumor xenografts. Boxplot representations of the collagen fiber densities as quantified from the lymph nodes are shown in Figure 3A. A one-way analysis of variance (ANOVA), followed by Fisher's least significant difference test (LSD) was calculated on all collagen densities in lymph nodes and is shown in Figure 3B and Supplementary Tables 1–2.

### Metastatic breast cancer up-regulates major ECM proteins in affected lymph nodes

**Figure 4:**
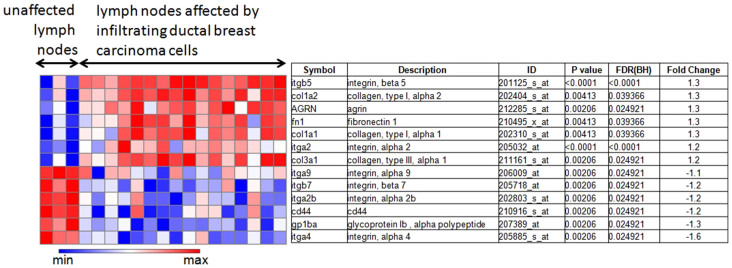
Gene expression changes in genes driving the interaction between the ECM and ECM receptors in human lymph nodes containing metastatic breast cancer cells. Microarray gene expression data were obtained from human lymph nodes affected by infiltrating ductal breast carcinoma cells as compared to unaffected lymph nodes. Total RNA was isolated and prepared for hybridization to human Affymetrix Gene Chip arrays (GSE44408). The heat map and corresponding statistical analysis was generated using the Gene-e matrix visualization and analysis platform (http://www.broadinstitute.org). FDR(BH) < 0.05 was considered significant.

We performed gene expression analysis on publicly available microarray data (GSE 44408) of lymph node samples from breast cancer patients. Human lymph nodes that contained metastasized breast cancer cells demonstrated an up-regulation of major ECM proteins, such as collagen, fibronectin, and several types of integrins as shown in Figure 4.

## Discussion

Here we have shown that sentinel lymph nodes from mice with metastatic breast tumors, but not with non-metastatic tumors, displayed significantly increased collagen fiber densities. This increase in collagen fibers is accompanied by a degeneration of the cortex structure of the lymph nodes. The increase in collagen fibers in lymph nodes of mice growing metastatic tumors may be caused by the cancer cells in these lymph nodes, which are known to recruit fibroblasts through cytokines and growth factors^21^. Signaling between the cancer cells in these lymph nodes and fibroblasts may have stimulated fibroblasts to produce more collagen^22^. It is also possible that breast cancer cells are able to produce collagen fibers on their own and thereby cause a fibrotic reaction that is independent of the host response^23^. The observed increases in collagen fiber density may play a role in facilitating additional circulating tumor cells to adhere to this collagen network in lymph nodes and form a secondary tumor. It is generally accepted that the lymphatic flow delivers tumor cells from the lymphatics into the draining lymph nodes^24^,^25^. However, it is not clear whether this is a controlled step. There have been some reports that chemokines such as CCL1 and CCL2 expressed by lymphatic sinuses in the lymph node may mediate the entry of tumor cells into the lymph node^24^.

The micro-architecture of the lymph node is largely supported by a reticular network of fibroblastic reticular cells, which produce collagens I, III and IV, elastin, entactin, fibronectin, laminin-1, tenascin, vitronectin, and heparan sulfate^26^. Reticular fibers are thought to trap metastasizing tumor cells, thereby resulting in the formation of metastatic colonies^27^. Surviving cancer cells in the lymphatic system may continue towards secondary tumor formation in lymph nodes with the support of the lymph node microenvironment^28^. Our findings suggest that the presence of metastatic breast cancer cells in the sentinel lymph nodes increases the density of collagen fibers and basement membranes and may be a necessary step during metastatic progression. Breast cancer cells were shown to move along aligned collagen I fibers^29^, and the increased density of collagen I fibers in lymph nodes containing metastatic breast cancer cells may facilitate the spread of these cancer cells along these fibers within the lymph node. It is likely that the increased collagen fiber content of lymph nodes containing metastatic breast cancer cells increases the stiffness of these lymph nodes, which in turn may affect overall cell and in particular cancer cell behavior in these lymph nodes^29^,^30^,^31^.

Studies have shown that the lymph nodes of athymic nude mice have intact B cell follicles and an inhibited development of T-lymphocytes^32^,^33^. A small population of T cells, a low T-dependent response, and an increased natural killer cell response are the characteristics of the immune system of athymic nude mice^38^. We observed a lack of B cell follicles in the sentinel lymph nodes of SUM159 tumor bearing mice and in the MDA-MB-231 tail-vein injected mice. A subset of fibroblastic reticular cells (FRCs) was shown to establish a favorable niche for B lymphocytes via production of the cytokine BAFF, which is a B cell activating factor^34^. Our data suggest that FRCs, through the presence of metastatic tumor cells in the lymph nodes, may lay down more collagen and at the same time may suffer from a change in the production of the cytokine BAFF, which in turn would reduce the number of B cell follicles. The lack of B cells in the lymph nodes would lead to a reduced capability of identifying antigens or pathogens that are circulating in the blood or lymph and thus would have harmful consequences on humoral immune responses^34^.

Our analysis of public domain human lymph node data indicated that chemokines such CCL1 and CCL2 may promote lymphatic extravasation and breast cancer metastasis to the sentinel lymph nodes^24^,^25^,^35^,^36^. A recent study showed that the entry of metastatic human breast cancer cells into lymph nodes is controlled by the CCL1 chemokine, which is expressed by the lymphatic sinuses of lymph nodes^24^. In a different study, CCL2 induced the expression of the intercellular adhesion molecule ICAM-1 on human lymphatic endothelial cells and thereby facilitated the attachment of breast cancer cells to lymphatic endothelial cells^36^.

Collagen fibers are an important component of the ECM, and increased stromal collagen in primary tumors can facilitate breast tumor formation, invasion, and metastasis^29^,^37^,^38^. A clinical pilot study revealed that primary clinical breast cancers that had metastasized to the lymph nodes contained significantly elevated levels of collagen fiber volume and significantly decreased inter-fiber distance^38^. However, the relationship between procollagen and collagen is not proportional^39^, and the interaction between fibroblasts and certain types of tumor cells can cause the fibroblasts to produce collagen^21^. We have performed gene expression analysis on publicly available microarray data (GSE16795) of human breast cancer cell lines where the breast cancer cell lines were grown to optimal cell densities for RNA extraction and hybridization on Affymetrix microarrays. The heat map as shown in Supplementary Figure 2 represents the changes in the relative content of type I pro-collagen alpha 1 chain (COL1A1) and type III procollagen alpha 1 chain (COL3A1) in 15 metastatic breast cancer cell lines and 11 non-metastatic breast cancer cell lines. The COL1A1 and COL3A1 gene expression levels are not significantly different.

Our focus on collagen fibers in sentinel lymph nodes is novel and suggests that collagen fiber density in sentinel lymph nodes may be used as a potential surrogate marker for assessing the presence of metastatic breast cancer cells in these lymph nodes. In the clinical setting, the number of involved lymph nodes in breast cancer patients predicts the overall outcome and the kind of treatment needed^40^. We have shown here that the ECM composition, and in particular the collagen fiber density, of lymph nodes may be an informative parameter for assessing the aggressiveness of a given breast cancer, which could be measured as part of the pathology work-up of the removed lymph nodes. Collagen fiber density can also be detected and quantitatively characterized by optical second harmonic generation (SHG) microscopy without requiring an exogenous optical imaging agent^38^. Clinical translation of the SHG-collagen fiber signature as a biomarker of metastatic potential may be possible as a minimally invasive fiber-optic approach that is coupled with lymph node biopsy. Imaging of the collagen fiber signature in lymph nodes may also allow for predicting drug delivery and breast cancer treatment outcome.

## Methods

### Cell culture

The human breast cancer cell lines MDA-MB-231, SUM159, T-47D, and MCF-7, were obtained from the American Type Culture Collection (ATCC, MD). Cells were stably transfected with a construct containing DNA of tdTomato as outlined in supplementary materials and methods and in Supplementary Figure 3. All cells were incubated at 37°C with 5% CO_2_ in a humidified incubator. Descriptions of cell specific culture media are given in supplementary materials and methods.

### Isolation and expansion of circulating breast cancer cells

The tdTomato-fluorescence was used to isolate and sort circulating tumor cells (CTCs) obtained from mice bearing orthotopic tdTomato-expressing MDA-MB-231 breast tumor xenografts. In brief, approximately 300 to 500 μl of blood was collected from the heart in a sterile Eppendorf tube. 10 to 20 units of heparin were added and mixed well to avoid blood clotting. The blood sample was then directly added to a cell culture flask containing standard cell culture medium. After two weeks in culture, tdTomato-expressing MDA-MB-231 CTCs were FACS sorted and further expanded.

### In vitro assays

Details of the protein extraction from cells and immunoblotting are provided in supplementary materials and methods.

### Experimental animal models

The Institutional Animal Care and Use Committee approved the animal protocol. Breast cancer cells (2 × 10^6^) were orthotopically implanted into the fourth right mammary fat pad of six-week-old female athymic nu/nu mice (NCI) as described previously^41^. Mice with BT-474, T-47D, and MCF-7 tumor xenografts were supplemented with 17β-Estradiol (Innovative Research of America, SE#121, 0.72 mg/pellet, 60 day release) in the neck region 24 hours prior to tumor inoculation^42^. Details for all tumor models and necropsy procedures are provided in supplementary materials and methods. The total number of lymph nodes per group was given in supplementary table 3. All experiments were carried out according to the approved guidelines of the Institutional Animal Care and Use Committees (IACUCs) of the Johns Hopkins University.

### Paraffin embedding, tissue sectioning, and hematoxylin-and-eosin staining

All tissues were paraffin-embedded, serially sectioned into 5 to 20 sections at 5 µm thickness, and mounted onto microscopy slides by the ‘Histology Laboratory’ Facility of the Molecular & Comparative Pathobiology Department of the Johns Hopkins Medical Institutions. Unstained sectioned were further processed for hematoxylin-and-eosin (H&E), Masson’s trichrome, periodic acid-schiff (PAS) and cytokeratin staining as detailed in supplementary materials and methods.

### Collagen density calculation

Tissue collagen density was calculated from the trichrome stained slides, using in-house software written in Matlab® (MathWorks, Natick, MA) as detailed in supplementary materials and methods. The PAS slides were analyzed visually and qualitatively for the presence of PAS positive stain.

### Microarray dataset

We used the publicly available microarray dataset GSE 44408 from the Gene Expression Omnibus (GEO) of the National Center for Biotechnology Information (NCBI) of the United States National Library of Medicine (http://www.ncbi.nlm.nih.gov/geo/), which contains data from human lymph nodes affected by breast cancer. These data were generated by total RNA extraction from 20–30 µm cryosections, which were hybridized to human Affymetrix GeneChip arrays, which are continuously updated whole-genome microarray chips^43^. 16 lymph node samples to which ductal breast carcinoma had metastasized, and 3 unaffected control lymph nodes were analyzed. The heat map and corresponding statistical analysis was generated using the Gene-e matrix visualization and analysis platform (http://www.broadinstitute.org). The false discovery rate (Benjamini–Hochberg) method was used to obtain adjusted P-values. FDR(BH) < 0.05 was considered significant.

### Statistical analysis

Box-and-whisker plots were generated by BoxPlotR^44^, where the center lines show the medians, box limits indicate the 25th and 75th percentiles as determined by the R software, whiskers extend 1.5 times the interquartile range from the 25th and 75^th^ percentiles, and outliers are represented by dots. A one-way analysis of variance (ANOVA) was calculated on all nodal collagen densities from mice of all experimental groups. Post-hoc comparisons using the Fisher Least Significant Difference (LSD) were explored to compare the mean of one group with the mean of another group. p < 0.05 was considered statistically significant and is indicated in all figures with a *.

## Author Contributions

A.R. and K.G. designed research; A.R., C.B. and A.K. performed experiments; A.R., C.B., A.K., M.C., L.J. and K.G. analyzed data; and A.R., Z.B. and K.G. wrote the manuscript. All authors discussed the results and reviewed the manuscript.

## Supplementary Material

Supplementary Information Supplementary materials and methods
