# Supplementary material for: Metastatic breast cancer cells in lymph nodes increase nodal collagen density
Source: Sci Rep. 2015 May 7;5:10002. doi: 10.1038/srep10002 (PMC4423440; doi:10.1038/srep10002)
Supplement: Supplementary Information — Supplementary materials and methods [file srep10002-s1.pdf]

Metastatic breast cancer cells in lymph nodes increase nodal collagen density

Asif Rizwan[1]; Camille Bulte[1,2], Anusha Kalaichelvan[1,3], Menglin Cheng[1], Balaji Krishnamachary[1], Zaver M. Bhujwalla[1,4], Lu Jiang[1], Kristine Glunde[1,4*]

[1]The Johns Hopkins University *In Vivo* Cellular and Molecular Imaging Center, Division of Cancer Imaging Research, The Russell H. Morgan Department of Radiology and Radiological Science, The Johns Hopkins University School of Medicine, Baltimore, Maryland.

[2]Department of Neuroscience, College of Literature, Science, and the Arts Honors Program, University of Michigan - Ann Arbor, Michigan.

[3]Department of Health Sciences, Western University, London, Ontario, Canada.

[4]The Johns Hopkins University School of Medicine, The Sidney Kimmel Comprehensive Cancer Center, Baltimore, Maryland.

<u>**Supplementary materials and methods**</u>

Cell lines

We generated constitutively tdTomato-expressing breast cancer cell lines from non-metastatic human MCF-7 (luminal A type), non-metastatic human T-47D (luminal A type), metastatic triple-negative human MDA-MB-231 (basal type), and metastatic human SUM159 (metaplastic type) breast cancer cells by using a lentiviral expression vector system. Briefly, lentiviral vector expressing the tdTomato fluorescent protein under the control of the pGK promoter along with the ΔR8.2 packaging vector and the vesicular stomatitis virus (VSV-G) envelope vector were used to transfect 293T cells. Forty-eight hours post-transfection, virions present in the cell culture media were collected and concentrated. Concentrated virus was used to transduce MCF-7, T-47D, MDA-MB-231, and SUM159 breast cancer cells in the presence of 8μg/ml of polybrene (Sigma, St. Louis, MO). This process was repeated three times to obtain maximum transduction efficiency. Further, to generate a pool with high intensity fluorescence, transduced cells were sorted by fluorescence-activated cell sorting (FACS) as described in our previous study [1]. Briefly, three weeks following the expansion of tdTomato-fluorescent cells, FACS was performed at The Johns Hopkins University Flow Cytometry Core Facility using a FACS Vantage SE (Becton Dickinson, Franklin Lakes, NJ) instrument. Fluorescence gating was done on the brightest cells, which were collected and then expanded. tdTomato protein expression was detected by fluorescence microscopy using a 20x objective attached to a Nikon inverted microscope, equipped with a filter set for 528 to 553 nm excitation and 600 to 660 nm emission and a Nikon COOLPIX digital camera (Nikon Instruments, Inc, Melville, NY). (Supplementary Figure 3)

Cell type specific media used for breast cancer cell lines

**Cell Line**	**Basal Medium**	**Additives***
MDA-MB-231	RPMI 1640 (Sigma-Aldrich)	10% FBS
SUM159	RPMI 1640 (Sigma-Aldrich)	10% FBS/Ins/Hyd
T-47D	RPMI 1640 (Sigma-Aldrich)	10% FBS/E2
MCF-7	RPMI 1640 (Sigma-Aldrich)	10% FBS/Ins

The following table shows the specific media used for each cell line.

** All media contain 100 U/ml penicillin, 100 µg/ml streptomycin. Ins = Insulin; Hyd = Hydrocortisone, E2 = beta-Estradiol*

Protein extraction and immunoblotting

Cell pellets were lysed with radioimmunoprecipitation assay (RIPA) buffer (catalog number 9806, Cell Signaling) and protease inhibitors cocktail (catalog number P8340, Sigma Aldrich). 5 to 10 mg of tissue from lymph nodes or other mouse organs were placed in round-bottom Eppendorf tubes and immersed in liquid nitrogen for snap freezing. The tissues were homogenized by pellet pestle (catalog number Z35994, Sigma Aldrich) on ice with 100 to 150 μl of RIPA buffer with protease inhibitors. Protein concentrations were determined using the Pierce bicinchoninic acid (BCA) assay (catalog number 23225, Thermo Scientific, Waltham, MA). A total of 10 μg of protein from each tissue was separated by electrophoresis using a NuPAGE 4-12% Bis-Tris Gradient Gel (Life technologies, Grand Island, NY) and transferred to a polyvinylidene difluoride (PVDF) membrane (BioRad, Hercules, CA). Membranes were blocked in TBST (Cell Signaling) containing 5% milk powder. Membranes were incubated with anti-wide spectrum cytokeratin antibody (catalog number ab9377, Abcam) at a 1:1,000 dilution for 16 hours at 4^0C, and washed for 1 hour in TBST. Bound primary antibodies were visualized with appropriate horseradish peroxidase-conjugated secondary antibodies against mouse (catalog number NA931V) or rabbit (catalog number NA934V, both from GE HealthCare Life Science, Pittsburgh, PA) using enhanced chemiluminescence reagent (Thermo Scientific, Waltham, MA). The primary and secondary antibodies were stripped off the membrane with Restore Western Blot Stripping Buffer (catalog number 21062, Thermo Scientific) and β-actin was probed with anti-β-actin antibody (catalog number A1978, Sigma Aldrich) at a 1:5,000 dilution for 16 hours at 4^0C. Visualization of β-actin bands was performed as described above for cytokeratin.

Breast tumor models in mice

Breast cancer cells were suspended at $2x10^7$ cells ml^{-1} in a 50:50 (v/v) solution of Hank's balanced salt solution and Matrigel. Mice were anesthetized and $2x10^6$ cells were injected into the into the 4th right mammary fat pad of homozygous athymic nude mice (NCI, Fredrick, MD) as previously described [2]. Groups of were orthotopically inoculated with tdTomato-expressing

MCF-7 (n=3), T-47D (n=4), MDA-MB-231 (n=4), or SUM159 (n=3) cells. Mice with T-47D or MCF-7 tumor xenografts were implanted with 0.72 mg, 60-day release pellets of 17β-estradiol (Innovative Research of America, SE#121) in the scruff region 24 hours before tumor inoculation [3]. Primary tumor volumes were calculated from caliper measurements of tumors using the formula for volume = (4/3) x 3.14 x $(\text{radius})^3$ = (3.14/6) x (a.b.c), where a, b, and c are the three measured orthogonal diameters [2]. Additionally, primary tumor growth and metastasis formation were optically tracked once or twice a week by tdTomato fluorescence imaging using a Xenogen IVIS 200 optical imaging system. All mice were sacrificed at 8 to 12 weeks following orthotopic inoculation when primary tumors reached sizes of 500-1000 mm^3. Blood from mice growing metastatic tdTomato-expressing MDA-MB-231 tumor xenografts was expanded in cell culture for two weeks. Pure cultures of CTCs were obtained by tdTomato-based FACS sorting and expansion. A separate cohort of 3 mice was used for orthotopic inoculation of these MDA-MB-231-derived CTCs. In addition, we injected 3 mice with $1x10^5$ MDA-MB-231 cells in 100 µl phosphate buffered saline (PBS) each in the tail vein, which were sacrificed at 12 weeks following injection. Furthermore, 5 mice were used as control with no tumor inoculation.

Necropsy

Mice were sacrificed and inguinal superficial sentinel lymph nodes that were draining the primary tumor were isolated as indicated in supplementary figure 4. Once located, we removed one to two lymph nodes by holding it with forceps and using surgical scissors to isolate it from surrounding other tissue [4]. All lymph nodes were washed in PBS, fixed in 10% formalin for 24 hours, and then placed in 70% ethanol. Similarly, other metastatic target organs such as lung, liver, brain, femur, tibia, other mammary glands, and non-target organs such as spleen and heart were also isolated, fixed, and processed as described above for lymph nodes.

Paraffin embedding, tissue sectioning, and hematoxylin-and-eosin staining

All tissues were paraffin-embedded, serially sectioned into 5 to 20 sections at 5 µm thickness, and mounted onto microscopy slides by the 'Histology Laboratory' Facility of the Molecular & Comparative Pathobiology Department of the Johns Hopkins Medical Institutions. Tissues were serially sectioned from the mid-section of the respective tissue at its maximum diameter. Hematoxylin and eosin (H&E) staining was performed by the 'Histology Laboratory' Facility as

well. The parenchyma of lymph nodes is divided into cortex and medulla. The outer cortex region of lymph nodes is composed of B cell containing follicles, follicular dendritic cells, and a T cell zone containing mostly T cells and dendritic cells. The inner medulla and medullary cords consist of dense lymphoid tissue, which is mainly composed of macrophages. In between the medullary cords are large sinusoids, with occasional plasma cells inside of them [5]. The sinusoids are small capillaries where the lymphatic fluid is received and circulated [5]. In the lymph nodes of athymic nude mice, which lack T cells, but do contain B cells, B cell follicles can be distinguished from other structures in the H&E images by their distinct circular shape [6].

Masson's Trichrome staining

For Masson's Trichrome staining, slides containing 5 μm thick sections of whole lymph nodes were first deparaffinized through a series of washes in xylene and ethanol, which comprised 3 washed of 3 min each in xylene, 3 washes of 3 min each in 100% ethanol, a wash of 3 min in 95% ethanol, a wash of 3 min in 80% ethanol, and a wash of 5 min in deionized water. Then, they were placed in Bouin's solution (catalog number HT10-1, Sigma Aldrich, St. Louis, MO) overnight. After washing the slides with tap water, they were stained with Weigert's Iron Hematoxylin solution (catalog number HT10-79, Sigma Aldrich) for 5 min. Then, they were stained with Biebrich Scarlet-Acid Fuchsin (catalog number HT15-1, Sigma Aldrich) for 5 min. Next, they were placed in Phosphomolybdic/Phosphotungstic Acid working solution, which consisted of a 1:1:2 (v/v/v) mixture of Phosphotungstic Acid Solution (catalog number HT15-2, Sigma Aldrich), Phosphomolybdic Acid Solution (catalog number HT15-3, Sigma Aldrich), and deionized water for 10 min. To stain collagen in blue, the slides were then stained with Aniline Blue solution (catalog number HT15-4, Sigma Aldrich) for 5 min, and finally placed in 1% acetic acid solution for 5 min. After that, they were dehydrated by 2 washes of 3 min each in 95% ethanol, 2 washes of 3 min each in 100% ethanol, and 3 washes of 5 min each in xylene, and left overnight in xylene. Masson's Trichrome is a universal stain for all collagens, with a preference for larger collagen fibers such as collagen I [7] and bone in blue, muscle fibers in red, cytoplasm in pink, and nuclei in black.

Periodic Acid - Schiff (PAS) staining

For PAS staining, slides with lymph node sections were first deparaffinized through a series of

washes in xylene and ethanol as described above for 'Masson's Trichrome staining'. Then the slides were immersed in Periodic Acid Solution (catalog number 395-1, Sigma Aldrich) for 5 min, followed by several washes with distilled water. Next, the slides were immersed in Schiff's Reagent (catalog number 395-2, Sigma Aldrich) for 15 minutes and then washed in running tap water for 5 minutes. Finally, the slides were counterstained with hematoxylin (catalog number GSH-3, Sigma Aldrich) for 90 sec, and then rinsed in tap water for 30 sec. PAS stains reticular fibers composed of collagen III as well as basement membranes consisting of collagen IV, laminin, and fibronectin in magenta. In addition, PAS staining can stain polysaccharides such as glycogen, and mucosubstances such as glycoproteins and glycolipidsglycogen. The PAS slides were analyzed visually and qualitatively for the presence of PAS positive stain.

Immunoperoxidase staining of cytokeratins

For immunoperoxidase staining of cytokeratins, slides containing lymph nodes were first deparaffinized through a series of washes in xylene and ethanol, which comprised 3 washes of 5 min each in xylene, 2 washes of 10 min each in 100% ethanol, 2 washes of 10 min each in 95% ethanol, and 2 washes of 5 min each in deionized water. Then, they were brought to a boil with citrate buffer (catalog number C-9999, Sigma Aldrich) for 20 min to unmask antigens. Next, they were washed in distilled water and then placed in 3% hydrogen peroxide (catalog number H1009, Sigma Aldrich) for 30 min. After being washed with Tris Buffered Saline (TBST, catalog number 9997, Cell Signaling, Boston, MA) containing Tween-20, the slides were placed in blocking buffer containing 5% goat serum (catalog number G9023, Sigma Aldrich), 1% bovine serium albumin (BSA, catalog number A7906, Sigma Aldrich), and 0.1% Triton X-100 (catalog number 100, Sigma Aldrich) in PBS for 60 min at room temperature. Then they were incubated with primary antibody against wide spectrum cytokeratin (catalog number ab9377, Abcam, Cambridge, MA) in a 1:200 dilution in blocking buffer at 4°C overnight. The next day, following a wash in TBST, the slides were covered with Signal Stain Boost Detection Reagent (catalog number 8114, Cell Signaling) and incubated at room temperature for 30 min. After a wash in TBST, the slides were covered with DAB Quanto Chromogen and Substrate (catalog number TA-060-QHDX, Thermo Scientific, Waltham, MA) for 5 min, and then washed with PBS. Finally, they were counterstained with hematoxylin for 1 min, washed in distilled water, and dehydrated through a series of washes in xylene and ethanol as described above for 'Masson's Trichrome Staining'. The slides were mounted with coverslips using Permount (catalog number SP15, Fisher Scientific, Waltham, MA). Control slides were stained keeping all the steps the same without adding primary antibody or Signal Boost Detection Reagent [8].

Collagen density calculation

Tissue collagen density was calculated using in-house software written in Matlab® (MathWorks, Natick, MA) from the entire histology slide. In brief, the JPEG images of Masson's Trichrome stained slides were converted into L*a*b* color (also known as CIELAB) space using the Matlab image processing toolbox (http://www.mathworks.com/products/demos/image/color_seg_lab/ipexfabric.html/). L*a*b* space consists of a luminosity layer 'L*' and chromaticity-layers 'a*' and 'b*'. 'a*' indicates where the color falls along the red-green axis, and 'b*' indicates where the color falls along the blue-yellow axis. A small representative region of 20 pixels was selected as sample area for each color such as background, red, pink, and blue from an image to calculate each sample's average color in 'a*b*' space. Then we categorized each pixel in the image file using the nearest neighbor rule. Non-node pixels were excluded from the calculations. For example, if the distance between a pixel and the blue color marker was the smallest, then the pixel would be labeled as blue pixel. Collagen stained in blue was segmented into a separate image. The collagen density was calculated from the ratio of pixels positive for collagen divided by the total number of pixels in the image.

References

1. Winnard, P.T., Jr., Kluth, J.B. & Raman, V. Noninvasive optical tracking of red fluorescent protein-expressing cancer cells in a model of metastatic breast cancer. *Neoplasia* **8**, 796-806 (2006).
2. Rizwan, A. *et al.* Relationships between LDH-A, Lactate and Metastases in 4T1 Breast Tumors. *Clin Cancer Res* (2013).
3. Yarden, R.I., Lauber, A.H., El-Ashry, D. & Chrysogelos, S.A. Bimodal regulation of epidermal growth factor receptor by estrogen in breast cancer cells. *Endocrinology* **137**, 2739-2747 (1996).
4. Mathieu, M. & Labrecque, N. Murine superficial lymph node surgery. *J Vis Exp*, e3444 (2012).
5. Kierszenbaum, A. & Tres, L. *Histology and Cell Biology: An Introduction to Pathology*, Edn. 3rd. (Elsevier / Mosby, Maryland Heights, Missouri; 2011).
6. Economopoulos, V., Noad, J.C., Krishnamoorthy, S., Rutt, B.K. & Foster, P.J. Comparing the MRI appearance of the lymph nodes and spleen in wild-type and immuno-deficient mouse strains. *PLoS One* **6**, e27508 (2011).
7. Harvey, J. Disorders of Bone Marrow, in *Veterinary Hematology: A Diagnostic Guide and Color Atlas*, Edn. 1st 266-267 (Saunders, 2012).
8. Burry, R.W. Controls for immunocytochemistry: an update. *J Histochem Cytochem* **59**, 6-12 (2010).

Supplementary Figure 1

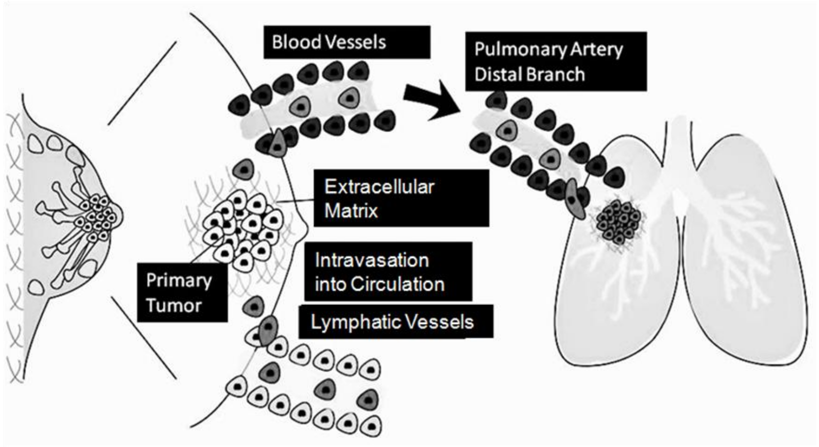

Supplementary Figure 1. Breast cancer metastasis to lymph nodes and distant organs. Breast cancer metastasis is a complex biological process with multiple steps. Detached cancer cells that are surviving and circulating in the blood or lymphatic vessels may get trapped in the lymph node or in the capillary beds of distant organs by adhering to endothelial basement membranes. (A) Schematic diagram where cancer cells can invade lymph nodes and blood vessels draining a primary breast tumor and spread to other parts of the body. (B) Schematic and color photograph of the sentinel lymph nodes in the mouse models used in our studies.

Supplementary Figure 2

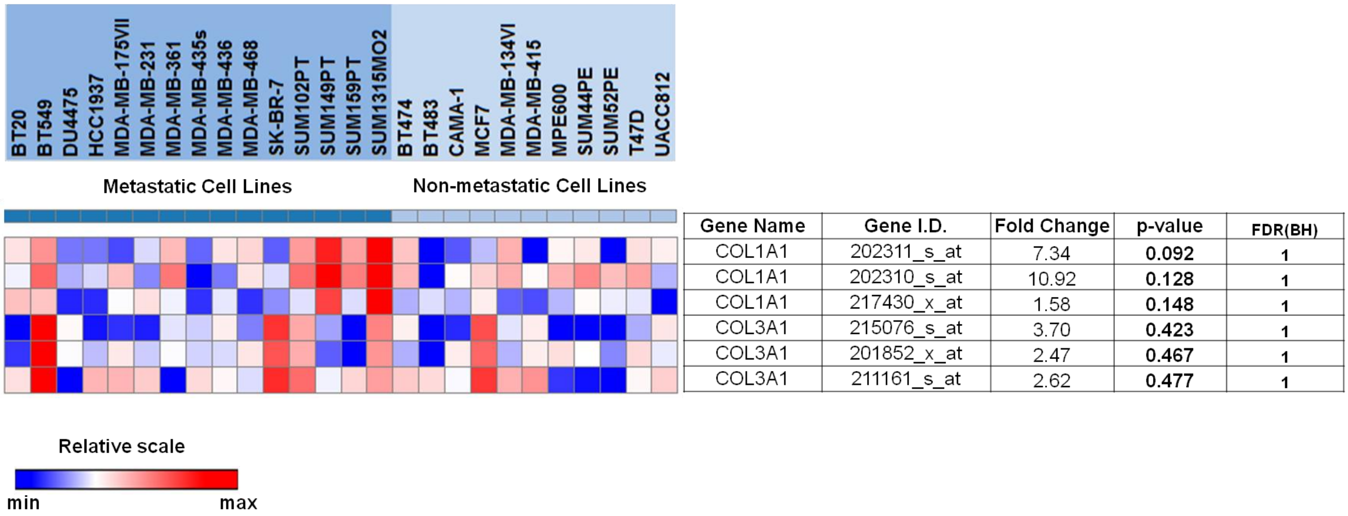

Gene Name	Gene I.D.	Fold Change	p-value	FDR(BH)
COL1A1	202311_s_at	7.34	**0.092**	**1**
COL1A1	202310_s_at	10.92	**0.128**	**1**
COL1A1	217430_x_at	1.58	**0.148**	**1**
COL3A1	215076_s_at	3.70	**0.423**	**1**
COL3A1	201852_x_at	2.47	**0.467**	**1**
COL3A1	211161_s_at	2.62	**0.477**	**1**

Supplementary Figure 2. Gene expression analysis of COL1A1 and COL3A1 in human breast cancer cell lines. This heat map represents the changes in the relative content of type I pro-collagen alpha 1 chain (COL1A1) and Type III procollagen alpha 1 chain (COL3A1) in 15 metastatic breast cancer cell lines and 11 non-metastatic breast cancer cell lines. The heat map was generated using the Gene-e matrix visualization and analysis platform (http://www.broadinstitute.org).

Supplementary Figure 3

A

Panel of metastatic and non-metastatic breast cancer cell lines and xenografts.
Metastatic lines in red and nonmetastatic lines are shown in blue.

Name	Type	ER	PR	ERBB2	Source	Disease	Metastases (Xenograft)
MDA-MB-231	Basal	-	-	-	Metastatic site	adenocarcinoma	Yes
SUM159PT	Basal	-	-	-	Primary breast tumor	anaplastic carcinoma	Yes
T47D	Luminal-A	+	+	-	Metastatic site	ductal carcinoma	No
MCF-7	Luminal-A	+	+	-	Metastatic site:	adenocarcinoma	No

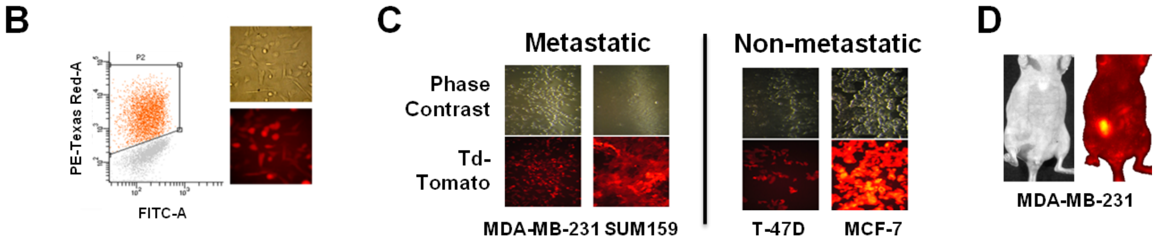

Supplementary Figure 3. tdTomato expressing breast cancer cell lines. (A) Panel of metastatic and non-metastatic breast cancer cell lines and xenografts. Metastatic lines are shown in red and non-metastatic lines are shown in blue. (B) Fluorescence gating used for FACS-sorting of tdTomato-expressing MDA-MB-231 cells (red). (C) Photographs of breast cancer cell lines expressing tdTomato *in vitro*. (D) Representative photographs of mice growing tdTomato-expressing breast tumor xenografts *in vivo*.

Supplementary Figure 4

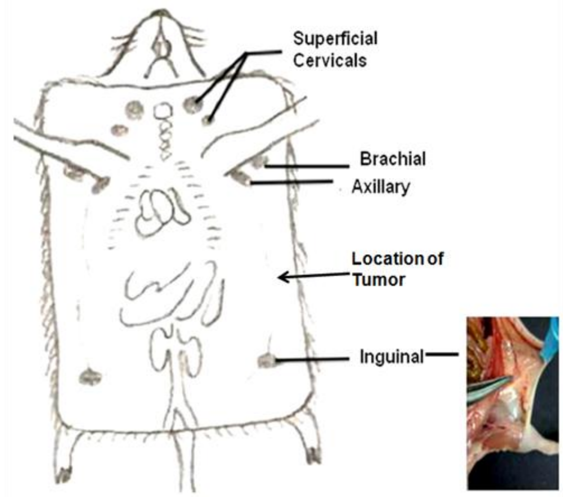

Supplementary Figure 4. Schematic and color photograph of the sentinel lymph nodes in the mouse models used in our studies.

Supplementary Table 1

One way ANOVA for collagen density in lymph nodes

Anova: Single Factor

SUMMARY

Groups	*Count*	*Sum*	*Average*	*Variance*
Control	8	20.35	2.54375	2.326741
MDA-MB-231	8	130.5	16.3125	38.92411
SUM159	6	80.8	13.46667	38.92667
T-47D	7	37.2	5.314286	5.084762
MCF-7	5	25.35	5.07	13.6395
Tail Vein-231	5	93.5	18.7	101.95
CTC-231-Tumor	5	80	16	12.5

ANOVA

Source of Variation	*SS*	*df*	*MS*	*F*	*P-value*	*F crit*
Between Groups	1651.881	6	275.3135	9.925983	1.59E-06	2.356179
Within Groups	1026.256	37	27.73664			
Total	2678.137	43				

Supplementary Table 2

Post-hoc LSD test for collagen density in lymph nodes

Difference between means

	Control	MDA-MB-231	SUM159	T-47D	MCF-7	Tail Vein-231	CTC-231-Tumor
Control	0.00	13.77	10.92	2.77	2.53	16.16	13.46
MDA-MB-231		0.00	2.85	11.00	11.24	2.39	0.31
SUM159			0.00	8.15	8.40	5.23	2.53
T-47D				0.00	0.24	13.39	10.69
MCF-7					0.00	13.63	10.93
Tail Vein-231						0.00	2.70
CTC-231-Tumor							0.00

Lease Significant Difference between groups: LSD= t(0.05/2, DFW) sqrt(MSW* (1/N1, 1/N2)

	Control	MDA-MB-231	SUM159	T-47D	MCF-7	Tail Vein-231	CTC-231-Tumor
Control	5.34	5.34	5.76	5.52	6.08	6.08	6.08
MDA-MB-231		5.34	5.76	5.52	6.08	6.08	6.08
SUM159			6.16	5.94	6.46	6.46	6.46
T-47D				5.70	6.25	6.25	6.25
MCF-7					6.75	6.75	6.75
Tail Vein-231						6.75	6.75
CTC-231-Tumor							6.75

Significant test : If (mean1-mean2)<LSD, then Not Significant

	Control	MDA-MB-231	SUM159	T-47D	MCF-7	Tail Vein-231	CTC-231-Tumor
Control		*	*	NS	NS	*	*
MDA-MB-231			NS	*	*	NS	NS
SUM159				*	*	NS	NS
T-47D					NS	*	*
MCF-7						*	*
Tail Vein-231							NS
CTC-231-Tumor							

NS= Not significant, *= Significant

Supplementary Table 3

	Number of lymph nodes per group						
	Control	MDA-MB-231	SUM159	T-47D	MCF-7	Tail Vein-231	CTC-231-Tumor
Total number of mice	5	4	3	4	3	3	3
Total number of lymph nodes	8	8	6	7	5	5	5
